# A signal-seeking Phase 2 study of olaparib and durvalumab in advanced solid cancers with homologous recombination repair gene alterations

**DOI:** 10.1038/s41416-023-02311-0

**Published:** 2023-06-26

**Authors:** Subotheni Thavaneswaran, Maya Kansara, Frank Lin, David Espinoza, John P. Grady, Chee Khoon Lee, Mandy L. Ballinger, Lucille Sebastian, Theresa Corpuz, Min Ru Qiu, Piyushkumar Mundra, Charles G. Bailey, Ulf Schmitz, John Simes, Anthony M. Joshua, David M. Thomas

**Affiliations:** 1 NHMRC Clinical Trials Centre, University of Sydney, Sydney, NSW Australia; 2 The Kinghorn Cancer Centre, St Vincent’s Hospital, Sydney, NSW Australia; 3 School of Clinical Medicine, Faculty of Medicine and Health, University of NSW, Sydney, NSW Australia; 4 Garvan Institute of Medical Research, Sydney, NSW Australia; 5 Kinghorn Centre for Clinical Genomics, Sydney, NSW Australia; 6 Department of Anatomical Pathology and Cancer Genetics, SydPath, St Vincent’s Hospital, Sydney, NSW Australia; 7 Cancer & Gene Regulation Laboratory Centenary Institute, The University of Sydney, Camperdown, NSW Australia; 8 Gene and Stem Cell Therapy Program Centenary Institute, The University of Sydney, Camperdown, NSW Australia; 9 Faculty of Medicine & Health, The University of Sydney, Sydney, NSW Australia; 10 Computational Biomedicine Lab Centenary Institute, The University of Sydney, Camperdown, NSW 2050 Australia; 11 Department of Molecular & Cell Biology, College of Public Health, Medical & Vet Sciences, James Cook University, Townsville, QLD Australia; 12 School of Biomedical Science, University of New South Wales, Sydney, NSW Australia

**Keywords:** Cancer genomics, Predictive markers

## Abstract

**Purpose:**

To determine the safety and efficacy of PARP plus PD-L1 inhibition (olaparib + durvalumab, O + D) in patients with advanced solid, predominantly rare cancers harbouring homologous recombination repair (HRR) defects.

**Patients and methods:**

In total, 48 patients were treated with O + D, 16 with *BRCA1/2* alterations (group 1) and 32 with other select HRR alterations (group 2). Overall, 32 (66%) patients had rare or less common cancers. The primary objective of this single-arm Phase II trial was a progression-free survival rate at 6 months (PFS6). Post hoc exploratory analyses were conducted on archival tumour tissue and serial bloods.

**Results:**

The PFS6 rate was 35% and 38% with durable objective tumour responses (OTR) in 3(19%) and 3(9%) in groups 1 and 2, respectively. Rare cancers achieving an OTR included cholangiocarcinoma, perivascular epithelioid cell (PEComa), neuroendocrine, gallbladder and endometrial cancer. O + D was safe, with five serious adverse events related to the study drug(s) in 3 (6%) patients. A higher proportion of CD38 high B cells in the blood and higher CD40 expression in tumour was prognostic of survival.

**Conclusions:**

O + D demonstrated no new toxicity concerns and yielded a clinically meaningful PFS6 rate and durable OTRs across several cancers with HRR defects, including rare cancers.

## Background

Homologous recombination repair (HRR) is comprised of interwoven pathways that function in the repair of DNA double-stranded breaks and interstrand crosslinks [1]. An inability to repair complex DNA damage and resolve DNA replication strain results in genomic instability and promotes cancer development [1]. BRCA1 and 2 are the best-characterised HRR genes and predispose to breast and ovarian cancer [2, 3]. Several additional genes have been implicated in this repair pathway and many studies have characterised the exploitation of these inherent defects for therapeutic opportunities [4–6].

The use of poly-ADP ribose polymerase (PARP) inhibitors has synthetically lethal activity in the presence of non-functional HRR. Olaparib monotherapy has yielded a prolonged progression-free survival in patients with relapsed platinum-sensitive high-grade serous ovarian cancer, particularly in the presence of germline or somatic *BRCA1/2* alteration [7–10]. Similarly, improved outcomes have been observed with olaparib across a range of cancer histotypes harbouring *BRCA1/2*, or other HRR gene alterations [5, 11–15]. Generally, patients with *BRCA* mutations derived greater benefits from olaparib treatment compared with other HRR gene alterations [16].

There is significant interest in the immunological consequences of DNA damage. Preclinical data suggest that DNA damage detected by the cyclic GMP-AMP synthase/stimulator of interferon genes (cGAS/STING) pathway triggers a proinflammatory cascade of cytokines [17]. Preclinically, PARP inhibition elicits a potent anti-tumour immune response through activation of the STING pathway in both BRCA1-deficient tumour cells and dendritic cells, possibly indicating an association between innate and systemic immunity [18, 19]. An incremental enhancement of BRCA1 and CD8 + T-cell-dependent anti-tumour effects has been observed with the addition of anti-programmed death (PD)-1 or PD-ligand1 (PD-L1) agents to PARP inhibition [18]. Taken together, there is a strong scientific rationale to prime these HRR-deficient tumours with PARP inhibition, expediting DNA damage, associated cell death and antigen release, in order to enhance response to immunotherapy [20, 21]. Several small clinical studies have examined the combination of PARP and programmed death (PD)-ligand1/PD-1 inhibition [22, 23]. A Phase I study of olaparib and durvalumab included two patients with advanced metastatic triple-negative breast cancer (TNBC) and 10 with high-grade serous ovarian cancer (HGSOC) [22]; 50% of each tumour type harbouring potentially pathogenic defects in the HRR pathway. One of the two patients achieving an objective tumour response (OTR) had *BRCA1* methylation, while the best response for the remaining patients with HRR-altered tumours was stable disease for at least four months. Grade 3 or higher adverse events (AE) included lymphopenia and anaemia and the only immune-related AE was grade 2 hypothyroidism [22]. The Phase 2 MEDIOLA study included genomically unselected baskets of gastric cancer, small cell lung cancer and germline *BRCA* carriers with metastatic breast cancer (NCT02734004). MEDIOLA reported a clinical benefit rate of 29% at 12 weeks, with no new safety concerns across the baskets [23]. Here we conducted a Phase II study to evaluate the safety and efficacy of olaparib and durvalumab (O + D) in patients with advanced solid and predominantly rare cancers, harbouring either somatic or germline BRCA1/2, or other HRR gene alterations.

## Patients and methods

### Study design and participants

This was a Phase II, open-label trial conducted at a single Australian centre (ACTRN12617001000392) within the framework of the Cancer Molecular Screening and Therapeutics (MoST) program [24]. Patients ≥18 years of age with treatment-refractory, locally advanced or metastatic cancers were screened using comprehensive genomic profiling of an archival tumour specimen. The panel-based assay employed for screening evolved over time and included in-house assays, Illumina TruSight Tumour 170, Illumina TSO500 and Foundation Medicine (FMI). Screening results were reviewed by a molecular tumour board (MTB) to match actionable genomic findings to relevant clinical trials of targeted therapies. Genomic eligibility for the trial was determined by the MTB and included pathogenic *BRCA1* or *BRCA2* alterations (excluding breast, ovarian and prostate cancers) or alterations in a range of prespecified HRR genes including *ATM, PALB2, RAD51C, RAD51D, CHEK1, CHEK2, ATR, CDK12, BAP1, BARD1, BRIP1* and *FANC*, group 2 (*n* = 32). The exclusion of breast, ovarian and prostate cancers was based on competing studies running concurrently in these histotypes and was also in line with our trial’s prioritisation of rare cancers.

Patients were required to have an Eastern Cooperative Oncology Group performance status (ECOG PS) 0–2; evaluable disease by Response Evaluation Criteria in Solid Tumours (RECIST v1.1) [25] and adequate hepatic, renal and bone marrow function. All patients needed to have failed (or be intolerant of) standard therapies for their tumour type, and not previously have received treatment with a PD-1, PD-L1, or a PARP inhibitor.

### Ethics approval and consent

The study was performed in accordance with the Declaration of Helsinki, with central or institutional ethics and local research governance approval. The MoST program has been approved by the St Vincent’s Hospital Sydney Human Research Ethics Committee (reference, HREC/16/SVH/23), as has this clinical trial. All participants provided written informed consent to partake in this study. An independent data and safety monitoring committee provided independent assessments of patient safety and trial progress.

### Study procedures

Eligible patients were enrolled into two groups based on the presence of *BRCA1 or BRCA2* alterations (*n* = 16, group 1) and other HRR gene alterations (*n* = 32, group 2), detailed in Table 1. All patients received olaparib, which was administered per oral at 300 mg twice daily on a continuous basis and commenced 28 days prior to the first dose of durvalumab. Patients also received a fixed dose of 1500 mg durvalumab as an intravenous infusion every 28 days for up to 13 cycles (starting day 1, cycle 2 of olaparib). Durvalumab ceased after a maximum of 13 cycles. Olaparib was continued until disease progression, unmanageable toxicity, or a decision by the patient or clinician to cease. Up to three dose reductions of olaparib and dose interruptions of both drugs were permitted for a maximum of 28 days on each occasion. Treatment toxicities were evaluated using the National Cancer Institute Common Terminology Criteria, version 4.03 [26]. Response assessment was performed every 8 weeks.

**Table 1:** Baseline characteristics and qualifying genomics by group.

Characteristic	Group 1 (*n* = 16)		Group 2 (*n* = 32)	
	No.	%	No.	%
Median age, years (range)	56 (23–71)		54 (20–76)	
Male sex	8	50%	13	41%
ECOG status				
0	15	94%	24	75%
1	1	6%	8	25%
Lines of prior systemic treatment				
Median line (range)	1.5 (1–3)		2 (0–5)	
Prior lines <2	8	50%	9	28%
Cancer type				
Bone and soft tissue sarcomas	4	25%	8	25%
Alveolar soft part sarcoma			1	3%
Chondrosarcoma			2	6%
Ewing’s sarcoma			1	3%
Leiomyosarcoma	2	13%	2	6%
Liposarcoma			2	6%
Osteosarcoma	1	6%	1	3%
PEComa	1	6%		
Carcinomas	12	75%	24	75%
Anal, SCC			1	3%
Breast, IDC			2	6%
Cervix adenocarcinoma	1	6%		
Cholangiocarcinoma, gallbladder adenocarcinoma	2	13%		
Colorectal adenocarcinoma	1	6%	3	9%
CUP			1	3%
Endometrial adenocarcinoma			2	6%
Ethmoid sinus adenocarcinoma	1	6%		
Gastric adenocarcinoma	1	6%	1	3%
Glioma	1	6%	2	6%
Medulloblastoma			1	3%
Meningioma, anaplastic			1	3%
Neuroendocrine carcinoma			1	3%
Ovarian adenocarcinoma			1	3%
Ovarian, sex cord-stromal tumour			1	3%
Pancreas adenocarcinoma	4	25%	2	6%
Small intestine adenocarcinoma	1	6%	1	3%
Thyroid carcinoma, papillary			1	3%
Uveal melanoma			2	6%
Qualifying genomic biomarker				
* BRCA1*	4^	25%		
* BRCA2*	12	75%		
* ATM*			10^#^	31%
* ATR*			1	3%
* BAP1*			2	6%
* BARD1*			1*	3%
* BRIP1*			2^$^	6%
* CDK12*			3^+^*	3%
* CHEK1*			1	3%
* CHEK2*			3	9%
* FANCA*			2*	6%
* FANCI*			1	3%
* NBN*			1	3%
* RAD51*			1	3%
* SLX4*			2*	6%
* XRCC2*			1	3%

*CUP* carcinoma of unknown primary, *HRR* homologous recombination repair, *IDC* infiltrating ductal carcinoma, *PEComa* perivascular epithelioid cell tumour, *SCC* squamous cell carcinoma.

*Indicates alterations that would not pass current bioinformatic pipelines, but were included in the original molecular tumour board report that qualified patients for the trial, comprised of 1 *BARD1, 1 CDK12, 1 FANCA, 1 ATM* + *CHEK1*, 1 *CHEK1* and both *SLX4* alterations. ^Two patients with a qualifying *BRCA1* alteration also had a co-occurring HRR gene alteration (1 in *ATM* and the other in *FANCD2*); ^#^four patients with qualifying ATM alterations also had co-occurring HRR gene alterations (2 in *CHEK1*, 1 in *NBN*, 1 in *CHEK1* and *BARD1* and 1 in *CHEK1* and *RAD51*); ^$^one patient with a qualifying *BRIP1* alteration also had a *RAD51C* mutation; ^+^two patients with qualifying *CDK12* alterations also had a *RAD51* mutation, or a *RAD51* and *FANCE* mutation.

### Endpoints

The primary endpoint was the clinical activity of O + D, as measured by progression-free survival at 6 months (PFS6). PFS6 is the Kaplan–Meier estimate of the proportion of patients who remain alive and progression-free at 6 months from the date of registration. Secondary endpoints included objective tumour response, overall survival (OS), ratio of time to progression (TTP) on trial (TTP2), to TTP on the last line of therapy (TTP1) prior to trial entry, health-related quality of life measured by the EORTC QLQ-C30 [27], and Brief Pain Inventory (BPI) assessment [28]. Response status was to be determined using RECIST version 1.1 or RANO guidelines at each assessment time point. Patients with an OTR (complete or partial response) will need confirmation of this response based on the results of the next scan. In a pan-cancer setting, using patients as their own control informs the rate of change in disease trajectory for that individual, with a TTP2:TTP1 ratio of 1.3 suggesting clinical activity [29, 30].

### Exploratory biological analyses

Where available, prior germline testing results were retrieved for all patients. When HRR alterations identified at screening met bioinformatic thresholds for potentially germline, germline testing was performed. Determination of a post hoc HRR defect score was planned but could not be undertaken due to tissue and sequencing assay limitations. Similarly, microsatellite instability status was also not evaluable.

Tumour mutational burden (TMB) has variable predictive capacity for immunotherapy benefit across a range of tumour types [31]. TMB was predominantly estimated according to the whitepaper methods outlined by Illumina [32], but with the removal of driver mutations (COSMIC count >1) in accordance with Lieber et al. [33] to reduce the ascertainment bias of sequencing known cancer genes. Due to the small panel size of the TST170 (0.56 Mb), we retained synonymous mutations in the TMB calculations, despite no expected contribution to neo-antigen production, but to increase the sample size of somatic mutations per patient and reduce noise in the calculation. There was also post hoc harmonisation across sequencing panels employed, where possible [34–36]. (Appendix 1A) and a TMB ≥ 10 mut/Mb used to define the high TMB group. The presence of co-occurring mutations, number of prior lines of treatment and platinum exposure was also evaluated with respect to clinical outcomes.

Tumour cell (PD-L1) expression was evaluated by immunohistochemistry using the Ventana PD-L1 (SP263) assay with cut-offs for positivity set at ≥1%. Archival tumour tissue was used to assess tumour infiltrating lymphocytes (TILs) and gene expression signatures corresponding to T-cell inflammation [37]. TILs were assessed using a hematoxylin and eosin-stained slide, which permitted morphological discrimination of lymphocytes in the tumour and its immediate periphery, as previously described [37–39]. The TILs level was quantified as proportion TILs, of total cells on a slide, and dichotomised as low and high using median TILs for the cohort. The NanoString nCounter was employed as a discovery research assay to examine RNA transcript levels and gene expression signatures of T-cell biology, inflammation and immune responses using a customised gene-set of 128 genes, including 5 housekeeping genes [40] (Appendix 1B).

The neutrophil:lymphocyte ratio (NLR) was determined using a baseline full blood count, defined as the ratio of absolute neutrophil count to absolute lymphocyte count, with a cut-off of four selected based on published thresholds for clinical benefit from immune checkpoint inhibition across various cancers [41].

Peripheral blood mononuclear cells (PBMC) collected at baseline, week 4 and week 8 were stained for immune markers of interest using multiparametric flow cytometry and screened for putative variables associated with treatment benefit. Data were acquired using LSR II Fortessa and FACS Diva software. Immunophenotyping data were analysed using FlowJo 199 (BD v10.6.2). For detailed information on optimised panels, refer to Appendix 1C; staining method and gating strategies (Appendix 1D). Post hoc analyses of clinical outcomes based on these biological characteristics was undertaken to assess their predictive and prognostic capacity. For the immune markers examined by flow cytometry, good responders were defined as those achieving an objective tumour response, or PFS >6 months and OS >24 months; all other patients were classified as poor responders. Due to the non-normality of the flow cytometry data, a Mann-Whitney test was used to assess immune cell populations in patients according to response. Survival analysis and Cox regression models used median values of cell populations of interest as the threshold for patient stratification. Tests were two-sided and *P* values ≤ 0.05 considered significant. A Benjamin–Hochberg correction was performed to minimise false discovery rates.

### Statistical considerations

This trial comprised of three modules of 16 patients each as defined by the MoST framework protocol [24]. One module included patients with *BRCA1* or *BRCA2* alterations (group 1) and two modules (based on the inclusion of 32 patients) consisted of patients with other HRR gene alterations (group 2). These groups were non-comparative. Prior to analysis (data lock for commencement of analysis: 12 March 2021), the primary objective of the trial was changed from a co-primary endpoint of OTR and TTP2/TTP1 to PFS6 within each group (amendment submitted: July 12, 2019); postulated to better reflect the clinical benefits of immunotherapy [42, 43]. A threshold for clinical activity using the PFS6 endpoint was however not set.

## Results

### Patient disposition and baseline characteristics

Of 162 molecularly eligible patients, 96 patients received a MoST MTB recommendation indicating trial eligibility. Forty-eight patients were subsequently enrolled on trial over a 15-month period (November 2017 and February 2019) and allocated to the two study cohorts (groups 1 and 2). Reasons for non-enrolment on trial are outlined in Appendix 2, with the most common reason being an excluded histotype.

Pancreatic adenocarcinomas (*n* = 4) and colorectal adenocarcinomas (*n* = 3) were the most common cancer types in group 1 and group 2, respectively, while 32 patients (66%) had rare or less common cancers. Group 1 had a median age of 55 years (range 23–72 years), 50% were male and 94% had an ECOG PS of 0. Molecularly, group 1 comprised of 12 patients with BRCA2 and 4 with BRCA1 alterations. Two patients had co-occurring HRR gene alterations and four qualifying alterations in group 1 were confirmed to have a germline origin. Group 2 had a median age of 54 years (range 20–76 years), 41% were male and 75% had an ECOG PS of 0. Molecularly, group 2 was comprised of a range of HRR alterations, with the most common being ATM (*n* = 9) and RAD51 (*n* = 5); 8 patients had >1 HRR alteration meeting molecular eligibility for the trial, four were confirmed to have a germline origin (Table 1).

### Primary clinical endpoint

**Fig. 1:**
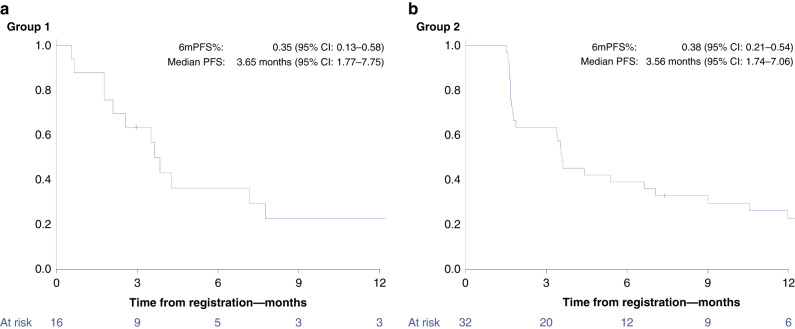
Kaplan–Meier curves for progression-free survival (PFS) by group. **a** PFS in group 1 (*BRCA1/2* alterations) and **b** PFS is group 2 (other homologous recombination repair alterations).

After a median follow-up period of 34 months, the PFS6 rate was 35% (95% confidence interval (CI), 13– 58%) in group 1 and 38% (95% CI 21–54%) in group 2. The median PFS was 3.7 (95% CI 1.8–7.8) and 3.6 months (95% CI 1.7–7.1) in group 1 and group 2, respectively. The Kaplan–Meier analyses are shown in Fig. 1.

### Secondary clinical endpoints

**Fig. 2:**
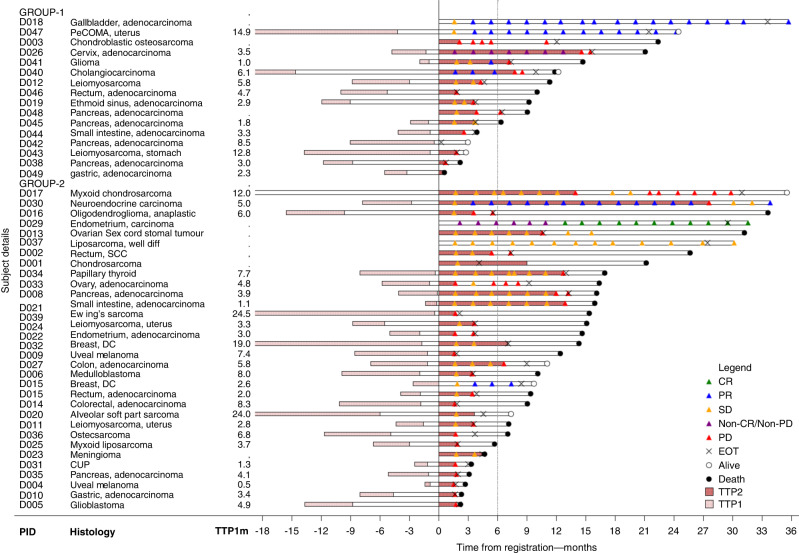
Swimmer plot characterising secondary trial endpoints by the individual patients within each group. Group 1: *BRCA1/2* alterations and Group 2: other homologous recombination repair alterations. TTP1—time to progression prior to trial, with the bar left of 0 depicting duration of therapy and timing of prior therapy in relation to commencing on trial. PID patient ID, CR complete response, PR partial response, SD stable disease, PD progressive disease, EOT end of treatment, TTP2 time to progression on trial.

Three patients in each group achieved an OTR, all partial responses in group 1 and two partial and one complete response in group 2, equating to an OTR of 19 and 9%, respectively. Amongst this subset of patients achieving an OTR, median PFS was 31.5 months and duration of response, 19.6 months. One patient in each group did not have measurable disease at baseline. Two other patients in group 1 had non-evaluable disease based on not undergoing any further imaging after trial enrolment. Five patients (31%) in group 1 achieved stable disease (SD) as best response, of whom one patient was progression-free at 6 months. In group 2, 18 patients (56%) achieved SD and 9 (28%) remained progression-free at 6 months. Seven patients (44%) in group 1 and 11 (34%) in group 2 had progressive disease as their best response, 13 confirmed radiologically and the others based on clinical progression (Table 2). To account for the heterogeneity of cancer types and natural histories, we calculated the TTP2/TTP1 ratio for 13 (group 1) and 28 patients (group 2), where TTP1 on therapy prior to study enrolment was evaluable (Appendix 3). A TTP2/TTP1 ratio of >1.3 (the pre-defined threshold for clinical activity) was achieved in 4 (31%) patients in group 1 and 7 (25%) patients in group 2 (Fig. 2). Three patients in group 1 and five in group 2 achieved a TTP2/TTP1 > 1.3 in the absence of an OTR. In both groups, all patients achieving an OTR, remained progression-free at 6 months. Of interest, amongst the 17 patients who met the primary PFS6 endpoint, 12 (70%) had an evaluable TTP ratio, with a median TTP2/1 ratio of 2.23 (range 0.37–11.2) and 8 (67%) achieving a TTP ratio>1.3, indicating an improved disease trajectory on study. The median OS was 11.3 months (95% CI 6.3–21.1) in group 1 and 15.1 months (95% CI 9.0–16.4) in group 2.

**Table 2:** Best response and time to progression (TTP) ratios.

Best response	Group 1 (*n* = 16)			Group 2 (*n* = 32)		
Complete response	0 (0%)			1 (6%)		
				D029	endometrial carcinoma	*CHEK2*
Partial response	3 (19%)			2 (6%)		
	D018	gallbladder, adenocarcinoma	*BRCA2*	D007	breast, IDC	*ATM*
	D040	cholangiocarcinoma	*BRCA2*	D030	NEC	*ATM*
	D047	PEComa	*BRCA2*			
Stable disease	5 (31%)			18 (56%)		
Non-CR/non-PD	1 (6%)			0 (0%)*		
	D026	cervix, adenocarcinoma	*BRCA2*			
Progressive disease	7 (43%)			11 (34%)		
Response maintained > 6 m	5 (31%)			12 (38%)		
	4 patients achieving PR, non-CR- non-PD +			3 patients achieving an objective response +		
	D041	glioma	*BRCA2*	D001	chondrosarcoma	*FANCA*
				D008	pancreas adenocarcinoma	*BRIP, RAD51*
				D013	ovarian sex cord-stromal tumour	*CHEK2*
				D017	chondrosarcoma	*ATR*
				D021	small intestine adenocarcinoma	*CDK12, RAD51*
				D027	colorectal adenocarcinoma	*RAD51*
				D032	breast, IDC	*ATM*
				D034	papillary thyroid	*ATM, NBN*
				D037	liposarcoma	*CDK12*
TTP2/TTP1 > 1.3	4 (31%)			7 (25%)		
	D026	Cervix, adenocarcinoma	*BRCA2*	D004	uveal melanoma	*BAP1*
	D041	Glioma	*BRCA2*	D007	breast, IDC	*ATM*
	D045	Pancreas, adenocarcinoma	*BRCA2*	D008	pancreas, adenocarcinoma	*BRIP, RAD51C*
	D047	PeCOMA, uterus	*BRCA2*	D015	colorectal, adenocarcinoma	*FANCI*
				D021	small intestine, adenocarcinoma	*CDK12, RAD51*
				D030	NEC	*ATM*
				D034	papillary thyroid	*NBN, ATM*

*IDC* infiltrating ductal carcinoma, *NEC* neuroendocrine carcinoma, *PEComa* perivascular epithelioid cell tumour.

Underlined study ID indicates patients who achieved both an objective response and TTP2/TTP1 > 1.3. *D029 had non-measurable disease at baseline but achieved a CR based on subsequent scans.

### Safety and tolerability

In terms of treatment exposure, four patients came off the study whilst receiving olaparib alone and did not commence durvalumab: two due to progressive disease and two due to adverse events. The median relative dose intensity (the ratio of administered doses to planned doses) was 100% (range 70–100%). The dose of olaparib was reduced in 8 patients (17%), with six of these patients also requiring treatment delays. These dose reductions were mainly due to anaemia, nausea and vomiting, or elevated creatinine. An additional 14 patients (29%) required delays in olaparib without dose reductions and four patients required a delay of durvalumab. At the time of study analysis, there were two patients in each group still receiving olaparib.

The most common AEs across grades were nausea (*n* = 29, 60%), anaemia (*n* = 21, 44%) and fatigue (*n* = 22, 46%). Grade 3 or worse AEs were reported in 21 patients (44%), comprised mainly of anaemia (8%), abdominal pain (6%), elevated amylase or lipase (6% each) and pancreatitis (4%). Fifteen serious AEs were experienced in 10 patients, with 5 (in 3 patients) adjudicated as related to study drug(s). Acute renal impairment was the only serious AE adjudicated as related to durvalumab (Appendix 4).

Compared to visit 1, no change was observed in mean on-study global health status based on the QLQ-C30, +1.3 (95% CI: −5.3 to +7.9) across all study participants. Importantly, no change was observed in mean global health status amongst 16 patients progressing before 6 months, +0.7 (95% CI: −9.7 to +11.1), while the 17 patients who remained progression-free at 6 months experienced a modest increase in mean global health status of +1.9 (95% CI: −7.4 to +11.2). Overall, none of these changes in global health status met thresholds for clinically meaningful differences [44].

### Predictors of response to PARP inhibition

Eight (17%) patients had ClinVar pathogenic/ likely pathogenic germline variants in *BRCA2* (*n* = 4), *ATM* (*n* = 2), *NBN* (*n* = 1) and *NBN* and *ATM* (*n* = 1) (Appendix 5). The presence of a germline HRR gene alteration was not associated with significant differences in PFS (HR 1.12, 95% CI 0.51–2.47, *P* = 0.77) or OS (HR 1.98, 95% CI 0.87–4.51, *P* = 0.10). An examination of the most frequent co-mutations revealed that *TP53* did not correlate with PFS or OS, but *KRAS* did, with its co-occurrence associated with a worse PFS (HR 2.52, 95% CI 1.13–5.63; *P* = 0.02) and OS (HR 2.77, 95% CI 1.21–6.35; *P* = 0.01). There was evidence to suggest that prior receipt of platinum chemotherapy was associated with PFS (HR 0.47, 95% CI 0.22–0.99, *P* = 0.04) and OS (HR 0.45, 95% CI 0.20–1.00, *P* = 0.04). However, the number of lines of prior therapy did not correlate with PFS and OS (Appendix 6).

### Predictors of response to immunotherapy

Fifteen (94%) in group 1 and 31 (97%) in group 2 had an evaluable TMB; 7 and 3 patients in group 1 and 2, respectively, had a high TMB of ≥10 mutations/megabase. In group 1 median PFS was 4.3 months for high, compared with 2.9 months for low TMB (HR 0.35, 95% CI 0.10–1.23; log-rank *P* = 0.09). In group 2, median PFS was 5.4 months for patients with high TMB compared with 3.6 months for low (HR 0.87, 95% CI 0.20–3.74; *P* = 0.85). Overall, median survival was 16.4 and 12.4 months for high (*n* = 10) versus low TMB (*n* = 36), respectively (HR 0.70, 95% CI 0.30–1.60; *P* = 0.39). Notably, 9 out of 10 with a high TMB had SD or better as best OTR, and 5 had either CR, PR, non-CR/PR or a TTP2/TTP1 ratio of >1.3. By contrast, of 16 patients (33%) with progressive disease as best OTR, 15 (94%) had a low TMB. These data are consistent with the significant contribution of durvalumab to clinical outcomes.

PD-L1 expression was available for 13 patients (81%) in group 1 and 29 patients (91%) in group 2. Median tumour PD-L1 expression was 1 and 0% for groups 1 and 2, respectively, with 44 and 41% meeting the PD-L1 expression cut-off of ≥1%. PD-L1 expression by group, or across study participants did not correlate with PFS (HR 1.11, 95% CI 0.58–2.14, *P* = 0.7) or OS (HR 1.67, 95% CI 0.83–3.35, *P* = 0.15), Appendix 6. TILs were evaluable for 11 and 26 patients in groups 1 and 2, respectively. By dichotomising the cohort at a median TILs of 1, 25% in group 1 and 16% in group 2 qualified as having high TILs. High TILs did not correlate with PFS or OS in each group, or the overall study population (Appendix 6).

**Fig. 3:**
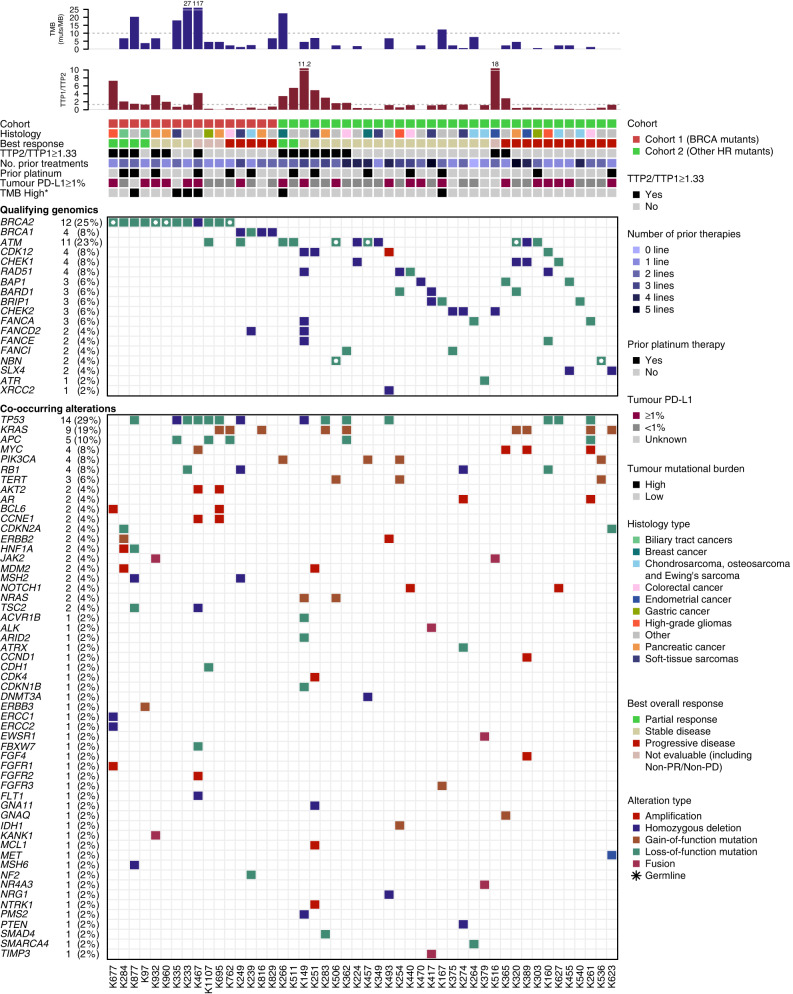
Detailed overview of the qualifying and co-occurring mutational profile for individual patients. Includes select clinical outcomes—best response, progression-free survival ≥ 6 months, time to progression 2 to time to progression 1 ratio, TTP2/TTP1 ≥ 1.3) by treatment group; clinical parameters—cancer histotype, no. of prior lines of treatment, prior platinum; and biomarkers of interest—genomic alteration type, tumour mutational burden, and PD-L1 status. HR homologous recombination repair, NLR neutrophil-lymphocyte ratio, PFS progression-free survival, TTP time to progression, TMB tumour mutational burden.

Exploratory analysis using an NLR threshold of 4 demonstrated that patients across both groups with an NLR ≥ 4 demonstrated a shorter OS (median 10.1 months) compared with NLR < 4 (median 15.3 months), HR for death 1.64 (95% CI 0.85–3.18; *P* value 0.14). The association of NLR with OS appeared to differ by group (interaction *P* = 0.05): HR for OS in group 1 was 0.54 (95% CI: 0.14–2.06; *P* = 0.37) and in group 2, 2.84 (95% CI: 1.22–6.63, *P* = 0.016) indicating a more pronounced effect of NLR on survival outcomes amongst patients with other HRR alterations (Appendix 6). There were no differences in median PFS observed. Figure 3 provides a detailed overview of the qualifying and co-occurring mutational profile of included cancer types alongside clinical outcomes and biomarkers of interest.

**Fig. 4:**
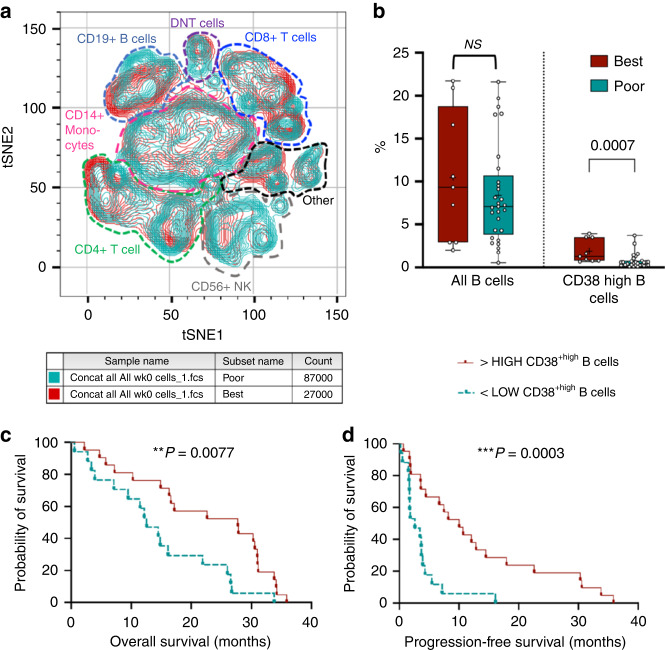
Immune subsets at baseline (week 0) that were associated with the best response. **a** Lymphoid panel, immune cell populations identified by Boolean gating were concatenated from all live PBMC samples at week 0 and analysed by t-SNE. Gated clusters poor (teal) and good (red) responders show the overlap of major immune lineages. **b** CD38^high^ B cells were associated with good (durable) responses. Analyses were done by Wilcoxon rank-sum tests with two-sided *P* values. **c**, **d** Kaplan–Meier curves showing better overall survival and progression-free survival of patients with PBMCs that have higher median levels of CD38^high^ B cells. Nominal *P* values shown are multiple-testing corrected using the Benjamin–Hochberg method.

We next used flow cytometry to assess a broad range of circulating immune biomarkers in peripheral blood at baseline, weeks 4 and 8, binned into good responders (OTR or PFS >6 months and OS ≥2 years), and poor responders. Figure 4a shows a t-SNE plot comparing good responders and poor responders across multiple hemopoietic lineages (further detailed in Appendix 7A). CD38 high B cells were associated with good response to O + D (Fig. 4b) and with longer OS and PFS (Fig. 4c, d and Appendix 7B). To differentiate prognostic and predictive effects, we assessed the survival of cancer populations expressing defining markers of B cells, CD38 and CD19 using publicly available data (GEPIA2). In multiple TCGA cohorts, including ovarian cancer, sarcomas and melanoma, CD38+ CD19+ correlated with better OS, suggesting a prognostic effect. Also, cell populations known to correlate with immunotherapy response (Appendix 7B), including T cells and PD-1 + B cells were seen at higher levels in baseline samples amongst good responders [45, 46]. OS and PFS also correlated with fewer circulating γδT cells and double-positive T cells, both likely immunosuppressive [47, 48].

We next sought evidence for an immune priming effect for olaparib that could explain response to combination treatment with O + D. Priming with olaparib decreased the proportion of all proliferating (Ki67+) T cells (*P* = 0.0049), CD4+ T cells (*P* = 0.0015), and B cells (*P* = 0.0026) from baseline to week 4. Olaparib did not alter the proportion of CD38+ B cells from baseline to week 4 (Appendix 8). Comparing the *BRCA1/2* cohort to the other HRR cohort, we observed differential IL-2 induction following priming of T cells ex vivo. Single-agent olaparib increased IL-2 production (a key cytokine in anti-tumour immunity) in T cells, which also trended with good responders (Appendix 9A). At week 8 following O + D we observed fewer cells expressing CD244 (a marker of immune exhaustion), while HLA-DR+ B cells were increased compared to baseline and was significantly associated with good responders (Appendix 9B). Experiencing a grade 3 or 4 immune-related AE (*n* = 6) was not associated with more favourable clinical outcomes (results not shown). Archival tumour tissue for RNA profile analysis was available for 39 patients and conducted using the NanoString nCounter. There was no clear association between the presence of an IFNγ signature, as previously shown by Ayers et al. and clinical outcomes in our study cohort [40]. The single-gene analysis found that higher CD40 expression in tumours (above median) was associated with longer OS (16.47 versus 9.17 months, *P* = 0.027).

## Discussion

In this signal-seeking trial, 14 of 48 patients (29%) achieved either an OTR or a favourable TTP ratio. This encouraging result fulfils the original co-primary endpoint of objective response and TTP2/1 >1.3.

O + D demonstrates a signal of clinical activity with a PFS6 rate of 35% (group 1) and 38% (group 2) across a range of tumour histotypes harbouring a HRR gene alteration. This is further supported by the durability of the objective tumour responses and an improvement in disease trajectory for individual patients, captured by the TTP2/1 ratios. Three patients (19%) achieved an OTR in group 1, along with three additional TTP2/1 evaluable patients (23%) who experienced a TTP2/1 ratio >1.3. In group 2, three patients (9%) achieved an OTR, and five additional patients (18%) demonstrated an improved TTP2/1 ratio. A median OS of 11.3 (group 1) and 15.1 (group 2) months is also favourable in a cohort comprised of rare cancers with poor prognoses. Combination treatment revealed no new toxicity concerns, with adverse events consistent with earlier trials [22, 23] and only one patient experiencing a SAE attributable to durvalumab. Exploratory biological analyses revealed that higher levels of baseline CD38+ B cells correlated with good clinical outcomes.

To our knowledge, this study is the first to demonstrate durable objective tumour responses to PARP plus PD-L1 inhibition in a gallbladder adenocarcinoma, cholangiocarcinoma, neuroendocrine carcinoma, a uterine perivascular epithelioid cell tumour (PeCOMA) and high-grade glioma, all selected on the basis of somatic or germline HRR gene alterations. Most of the cancers where we observed an OTR have limited therapeutic options beyond the first-line setting. Median OS for patients with locally advanced or metastatic cholangiocarcinoma and  gallbladder cancers treated with cisplatin-gemcitabine, or gemcitabine alone is only 11.7 and 8.1 months, respectively [49]. The addition of durvalumab to cisplatin-gemcitabine was recently reported in the TOPAZ-1 study, yielding an improved median OS of 12.8 months, (HR 0.80, 95% CI, 0.66–0.97; *P* = 0.021) compared with chemotherapy alone [50]. It would be interesting to see whether the subset of patients with HRR alterations derived a greater benefit from the platinum regimen, as well as an incremental benefit with the addition of durvalumab. While TOPAZ-1 may indicate a role for immunotherapy in the treatment of advanced biliary tract cancers, the data is limited for these other rare cancers [50–52]. A patient with a progressive and heavily pre-treated high-grade diffuse midline glioma achieved disease stabilisation for over 8 months, with an unconfirmed PR by RANO criteria.

Without randomisation, it is not possible to separate out the role of PARP inhibition and/ or PD-L1 inhibition in these durable objective responses and/or disease stabilisation. Earlier trials examining the safety and efficacy of PARP plus PD-L1 have mostly been histotype-specific and/or limited in their molecular eligibility. In the MEDIOLA basket studies, all cohorts were histotype-limited, with the advanced breast and relapsed ovarian cancer cohorts permitting only germline *BRCA1/2* alterations and platinum-sensitive disease [53, 54]. Even these studies of single histologies have not been able to ascertain the incremental value of combination treatment over PARP inhibition alone due to the absence of a comparator arm, limiting the available evidence to inadequate cross-study comparisons. In the advanced HER2-negative breast cancer setting, patients with germline *BRCA* mutations achieved a 60% response rate, median PFS of 7.0 months and OS of 19.3 months with PARP inhibition alone [12]. This was quite comparable to the MEDIOLA breast cancer cohort, who achieved an objective response rate of 63%, median PFS of 8.2 months and OS of 21.5 months with combination treatment [53]. The recently published JAVELIN PARP and JAVELIN BRCA/ATM studies were also non-randomised trials, evaluating combination treatment with immunotherapy and PARP inhibition, but using avelumab and talazoparib [55, 56]. The JAVELIN PARP study had DNA damage response positive (DDR+) cohorts mainly for histotypes that were excluded from our trial (breast, ovarian and prostate cancers) and otherwise comprised only three non-small cell lung cancer patients verified to be DDR+, amongst whom there were no objective responses [55]. In the JAVELIN BRCA/ATM study, a pan-cancer approach was taken, with patients selected on the presence of pathogenic *BRCA1/2* or *ATM* alterations. The objective response rate (ORR) in the BRCA1/2 and ATM cohorts was 26.4% and 4.9%, respectively, neither of which met the prespecified ORR of 40% for the trial [56]. Outside of histotypes excluded from our trial, OTR was seen in two pancreas cancers, three uterine leiomyosarcomas, one uterine sarcoma, one uterine carcinoma and testicular cancer. There were no OTR achieved amongst the ten cholangiocarcinoma or gallbladder cancers treated in the trial [56]. The higher OTR in the *BRCA1/2* group of this trial compared to ours may be due to differences in the included histotypes.

The first randomised trial of O + D versus durvalumab alone was published recently [57]. Amongst patients with untreated, platinum-ineligible metastatic urothelial carcinoma (*n* = 154), there was no demonstrable improvement in PFS or OS with the addition of olaparib to immunotherapy overall. However, amongst the subset of patients with HRR defects (*n* = 30), median PFS improved to 5.6 months (95% CI, 1.9–8.1) for patients receiving combination treatment, compared to 1.8 months (95% CI, 1.7–2.2) when treated with durvalumab alone (HR, 0.18; 95% CI, 0.06–0.47). While several HRR alterations were included as a stratification factor, only *ATM* (8.5%) and *BRCA2* (4.6%) alterations were identified amongst participants [57]. In the subset of patients with a HRR defect who received O + D treatment, 35% achieved an objective response with a median response duration of 6.7 months. While our cohort yielded a lower rate of objective responses, patients who achieved OTR experienced a median response duration of 19.6 months and a median PFS of 31.5 months. Although our study was underpowered to detect any synergy between these two therapies, we speculate that the higher OTR in group 1 suggests that PARP inhibition may contribute to tumour shrinkage, while the durability of the responses across both groups and possible association with TMB status suggests an effect of immunotherapy.

All objective responses in this trial were in tumours harbouring *BRCA2, ATM* or *CHEK2* alterations. This is consistent with the PROfound study results, where olaparib benefit was mainly limited to patients with *BRCA1* or *2* mutations [15]. Pre-defined immune biomarkers including tumour PD-L1, TILs and TMB did not strongly correlate with clinical outcomes and did not provide any insights into the incremental value of adding a PD-L1 inhibitor to PARP inhibition. Low baseline NLR levels was associated with a longer OS but not PFS, favouring a prognostic rather than predictive capability. Similarly, the poor prognostic implications of a *KRAS* mutation is more a reflection of the underlying cancer types (5 of 10 being pancreas cancer) as is potentially, the better outcomes associated with prior platinum therapy.

Exploratory biological studies compared baseline to week 4 samples after olaparib exposure alone, and then following exposure to O + D. The correlation of high baseline CD38+ B-cell populations with improved clinical outcomes is novel, although we did not observe an effect of olaparib priming on this cell population and cannot delineate histotype-based associations with high baseline levels. B cells can promote anti-tumour immunity through the release of cytokines such as IL-12, IFNγ, granzyme B and TRAIL57 [58]. The CD38+ B cells are likely transitional B cells or plasmablasts, however further characterisation is needed to determine the exact subset and function, with a view for therapeutic targeting. Interestingly, monoclonal antibodies (such as darartumumab) targeting CD38 lead to cell apoptosis. While they are already in clinical use for the treatment of multiple myeloma, the presence of CD38+ B cells was associated with good clinical outcomes with O + D, and therefore inhibition in solid cancers may not be desirable. Higher expression of CD40 in the tumour also correlated with good outcomes. CD40 is an important costimulatory molecule for B cells as well as dendritic cells, monocytes and other antigen-presenting cells [58]. CD40 agonists have been shown to trigger anti-tumour immune effects [59, 60] and combination treatment with olaparib and/or anti-PD-L1 antibodies may be a novel strategy in cancers with HRR defects.

The strengths of this signal-seeking study are inclusion of less common cancer histotypes and a rich range of correlative biomarkers that add depth to understanding individual responses. As rare and less common cancers account for half of all cancer deaths, their inclusion in clinical trials is vital to improving survival outcomes for patients with cancer as a whole. This however does introduce heterogeneity into the study population based on inherent differences in prognosis between cancer types. Also, the use of archival tissue for dynamic biomarker evaluation can challenge their interpretation.

## Conclusion

In sum, this study demonstrates a modest signal of activity for olaparib and durvalumab in understudied cancer populations. Clinical benefit was greatest amongst patients with *BRCA2* and *ATM* mutations, including durable objective tumour responses, with minimal toxicities attributable to combining olaparib with durvalumab. Amalgamating data from similar biomarker-driven trials, such as TAPUR and DRUP, will be critical to understanding histotype-specific outcomes in rare cancer populations.

## Supplementary information

Appendix

## Data Availability

The molecular data that forms the basis for MTB recommendations are available upon reasonable request. The authors declare that the data supporting the findings of this study are available within the paper and its supplementary appendix.
