# Supplementary material for: A signal-seeking Phase 2 study of olaparib and durvalumab in advanced solid cancers with homologous recombination repair gene alterations
Source: Br J Cancer. 2023 Jun 26;129(3):475–85. doi: 10.1038/s41416-023-02311-0 (PMC10403555; doi:10.1038/s41416-023-02311-0)
Supplement: Supplementary file 1 — Appendix [file 41416_2023_2311_MOESM1_ESM.pdf]

Appendices

Appendix 1. Biological correlates

A. Tumour mutational burden (TMB) methods

For the CCPv2/2.2 panel, sufficient information was not available to harmonise the TMB measures. Therefore, the 10 mutations/ megabase threshold was applied to the TMB value obtained from the CCPv2/2.2 assay. This identified only one high TMB (12.3 mutations/ megabase) amongst patients sequenced on this assay.

For TST170, the number of non-synonymous mutations/ megabase was calculated by normalising the WES-TMB value to 35 megabases according to method by Endris V et al (Int J Cancer. 2018), before further conversion to the FMI-equivalent.

Supplementary Table 1. Harmonisation of TMB measurements across sequencing panels

Substudy Patient ID	Sequencing assay	TMB at time of study analysis (muts/Mb)	Adjusted FMI-equivalent TMB (muts/ Mb)	Final TMB grouping across panels
D001	CCPv2.2	7.6	n/a	L
D002	CCPv2	12.3	n/a	H
D003	CCPv2.2	2.5	n/a	L
D004	TST170	2.3	4.3	L
D005	CCPv2	0	n/a	L
D006	CCPv2.2	1.9	n/a	L
D007	TST170	22.5	34.0	H
D008	TST170	0	0.9	L
D009	TST170	2.3	4.3	L
D010	CCPv2.2	0.6	n/a	L
D011	CCPv2.2	0.6	n/a	L
D012	TST170	18	27.4	H
D013	TST170	2.3	4.3	L
D014	CCPv2.2	1.3	n/a	L
D015	TST170	0	0.9	L
D016	TST170	0	0.9	L
D017	TST170	0	0.9	L
D018	TST170	6.8	10.9	H
D019	TST170	27	40.6	H
D020	TST170	0	0.9	L
D021	CCPv2	4.5	n/a	L
D022	TST170	0	0.9	L

D023	TST170	2.3	4.3	L
D024	TST170	0	0.9	L
D025	TST170	0	0.9	L
D026	TST170	117	172.9	H
D027	TST170	0	0.9	L
D029	TST170	0	0.9	L
D030	TST170	0	0.9	L
D031	TST170	0	0.9	L
D032	TST170	0	0.9	L
D033	TST170	6.8	10.9	H
D034	TST170	2.3	4.3	L
D035	TST170	4.5	7.6	L
D036	TST170	2.3	4.3	L
D037	CCPv2.2	7	n/a	L
D038	Genomics for Life			
D039	TST170			
D040	CCPv2	3.8	n/a	L
D041	TST170	0	0.9	L
D042	TST170	4.5	7.6	L
D043	CCPv2.2	1.3	2.8	L
D044	TST170	6.8	10.9	H
D045	TST170	0	0.9	L
D046	TST170	2.3	4.3	L
D047	TST170	20.3	30.8	H
D048	TST170	6.8	10.9	H
D049	TST170	4.5	7.6	L

Shaded cells indicate unavailable data. **Abbreviations:** Muts/Mb – mutations per megabase.

B. NanoString methods

Gene expression signatures required total RNA to be isolated from 4 μM-thick FFPE scrolls using the RNeasy DPS FFPE kit (Qiagen), following the manufacturer's protocol. RNA was quantified using a Qubit fluorometer (Thermo Fisher). Gene expression analysis was performed using the NanoString nCounter expression platform (NanoString Technologies)[1]. A custom codeset of 128 genes, including 123 genes related to T-cell biology, inflammation, and immune response and 5 housekeeping genes was applied[2-4]. For each sample, 150 ng of RNA in a total volume of 5 μl was mixed with 3' biotinylated capture probe and 5' reporter probe tagged with a fluorescent barcode. Probes and RNA were hybridized overnight at 65°C for 24 hours. Samples were run on a NanoString MAX preparation station, with removal of excess capture and reporter probes and loaded into the sample cartridge. Samples were scanned on the nCounter digital analyzer. Raw gene expression data was matched with the

Nanostring reference library file and then normalized using the Removing Unwanted Variation-III (RUV-III) method [1]. All statistical analyses have been performed in R (version 4.1) including data integration and visualisation using tidyverse (v 1.3.1), and survival analysis using survminer (v 0.4.9).

1. Molania R, Gagnon-Bartsch JA, Dobrovic A, Speed TP. A new normalization for Nanostring nCounter gene expression data. Nucleic Acids Res. 2019;47(12):6073-83. Epub 2019/05/23. doi: 10.1093/nar/gkz433. PubMed PMID: 31114909.
2. Ji, R. R. et al. An immune-active tumor microenvironment favors clinical response to ipilimumab. Cancer immunology, immunotherapy: CII 61, 1019-1031, doi:10.1007/s00262-011-1172-6 (2012).
3. Herbst, R. S. et al. Predictive correlates of response to the anti-PD-L1 antibody MPDL3280A in cancer patients. Nature 515, 563-567, doi:10.1038/nature14011 (2014).
4. Ayers, M. et al. Relationship between immune gene signatures and clinical response to PD-1 blockade with pembrolizumab (MK-3475) in patients with advanced solid tumors. Journal for Immunotherapy of Cancer 3, P80-P80, doi:10.1186/2051-1426-3-S2-P80 (2015).

C. Optimised antibody panels

Lymphoid+ panel

#	marker/reagent	fluorophore	clone	source	Phenotyping and state
1	FVS700	-	-	BD	
-	human Fc block	-	-	BD	
2	CD3	Brilliant Violet-786	UCHT1	BD	Total T-cells
3	CD4	Brilliant UV-737	SK3	BD	CD4+ T-cells
4	CD8a	Brilliant UV-395	RPA-T8	BD	CD8+ T-cells
5	CD14	Brilliant Violet-650	M5E2	BD	Monocyte subset macrophage
6	CD19	Brilliant Violet-711	SJ25C1	BD	Total B-cells
7	CD56	PE-Cy5	B159	BD	NKT cells, T-cell subsets
8	HLA-DR	Brilliant Violet-605	G46-6	BD	Activation
9	CD127	Brilliant Violet-421	HIL-7R-M21	BD	T-cell subsets
10	CD45RA	Brilliant Blue-515	HI100	BD	Naïve memory cells
11	PD-L1	PE	MIH1	BD	Checkpoint
12	CCR7	PE-CF594	150503	BD	CD197 (Naïve memory cells)
13	CD25	PE-Cy7	M-A251	BD	T-reg, activation
14	Va24Ja18	APC	6B11	BioLegend	TCR specific
15	CD38	APC-H7	HB7	BD	Naïve /memory
16	PD-1	Brilliant Blue-700	EH12.1	BD	Checkpoint
-	TF Fixation Buffer	-	-	BD	
-	TF Perm/Wash buffer	-	-	BD	
17	Ki-67	Brilliant Violet-486	B56	BD	Proliferation

Mixed/myeloid panel

#	marker/reagent	fluorophore	clone	source	
1	FVS700	-	-	BD	
-	human Fc block	-	-	BD	
2	CD3	Brilliant Violet-786	UCHT1	BD	Total T-cells
3	CD14	Brilliant Violet-650	M5E2	BD	Monocyte subset macrophage
4	CD19	Brilliant Violet-711	SJ25C1	BD	Total B-cells
5	CD16	APC-H7	3G8	BD	Neutrophils
6	CD56	PE-Cy5	B159	BD	NKT cells, T-cell subsets
7	CD11c	Brilliant Violet-480	B-ly6	BD	Monocytes, macrophages, Dc's
8	HLA-DR	Brilliant Violet-605	G46-6	BD	Antigen presentation
9	CD27	Brilliant Violet-421	M-T271	BD	Maturation
10	CD11b	PE	ICRF44	BD	Monocyte, macrophages
11	CD123	PE-Cy7	7G3	BD	Plasmacytoid Dcs
12	CD141	APC	1A4	BD	Myeloid Dcs (cDC1)
13	CD1c	Brilliant Blue-515	F10/21A3	BD	Myeloid Dcs (cDC2)
14	CD15	Brilliant UV-395	HI-98	BD	Neutraphils
15	CD244	PE-CF594	2B4	BD	NK cell, Dcs, MDSCs
-	2% pfa/FC buffer	-	-		

Intracellular cytokines panel

#	marker/reagent	fluorophore	clone	source	
1	FVS700	-	-	BD	
-	human Fc block	-	-	BD	
2	CD3	Brilliant Violet-786	UCHT1	BD	Total T-cells
3	CD4	Brilliant UV-737	SK3	BD	CD4+ T-cells
4	CD8a	Brilliant UV-395	RPA-T8	BD	CD8+ T-cells
5	CD14	Brilliant Violet-650	M5E2	BD	Monocyte subset macrophage
6	CD19	Brilliant Violet-711	SJ25C1	BD	Total B-cells
7	CD56	PE-Cy5	B159	BD	NKT cells, T-cell subsets
8	CD16	APC-H7	3G8	BD	Neutrophils
9	Va24Ja18	Brilliant Violet-421	6B11	BioLegend	TCR specific
10	CD11c	Brilliant Violet-480	B-ly6	BD	Monocytes, macrophages, Dc's
11	CD11b	Brilliant Violet-605	ICRF44	BD	Monocyte, macrophages
12	Va7.2	PE/Dazzle-594	3C10	BioLegend	Mait cells
13	gdTCR	PE-Cy7	11F2	BD	gd T-cells
-	Cytofix buffer	-	-	BD	
-	Cytoperm/wash buffer	-	-	BD	
14	IL-2	PE	MQ1-17H12	BD	Cytokine
15	IFNg	APC	B27	BD	Cytokine
16	TNFa	FITC	MAb11	BD	Cytokine

Note: PDL1 antibody M1H1 does not compete with durvalumab.

D. Flow cytometry gating strategy

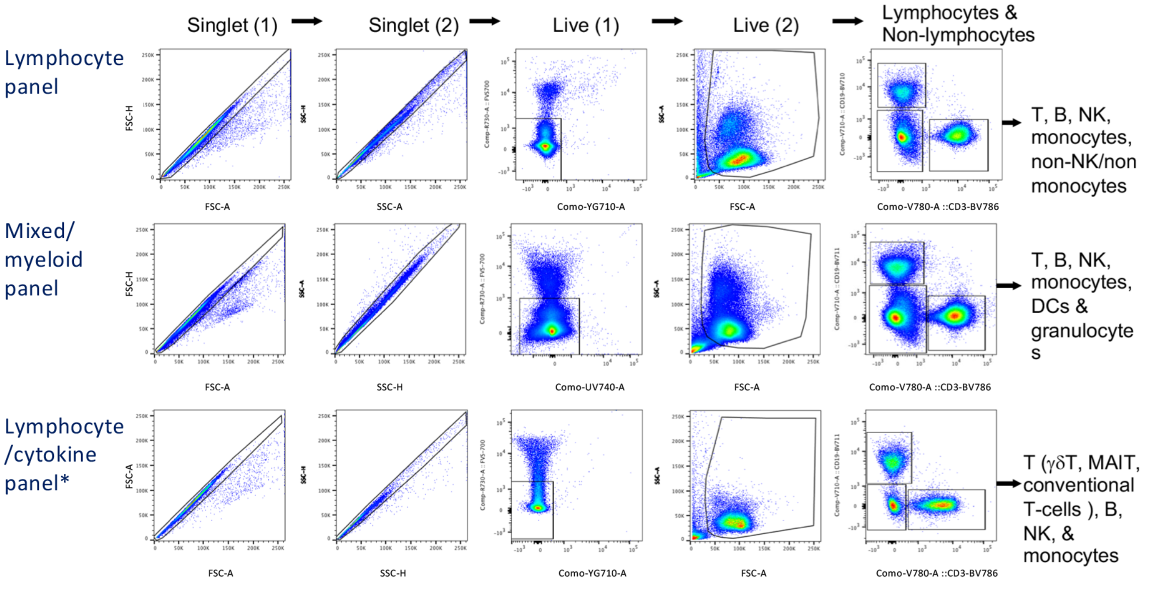

* No stimulant + Golgi plug and 4 hr culture, or PMA + Ionomycin + Golgi plug and 4 hr culture,

Figure 1. Gating CD3|CD19

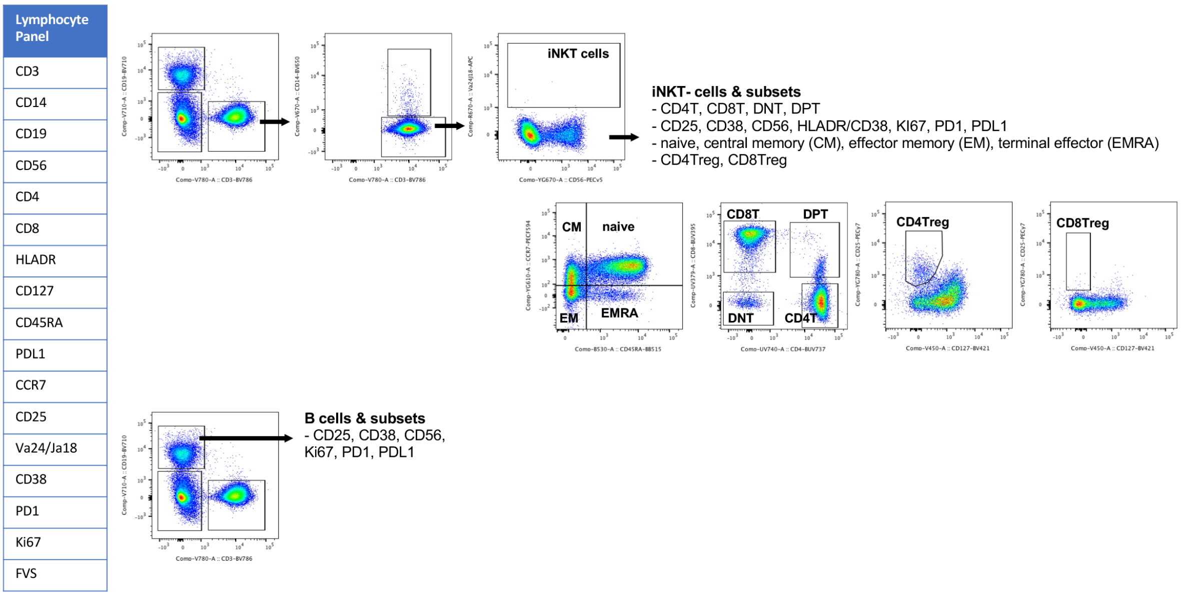

Figure 2. Gating T-cell panel from CD3|CD19

Lymphocyte Panel
CD3
CD14
CD19
CD56
CD4
CD8
HLADR
CD127
CD45RA
PDL1
CCR7
CD25
Va24/Ja18
CD38
PD1
Ki67
FVS

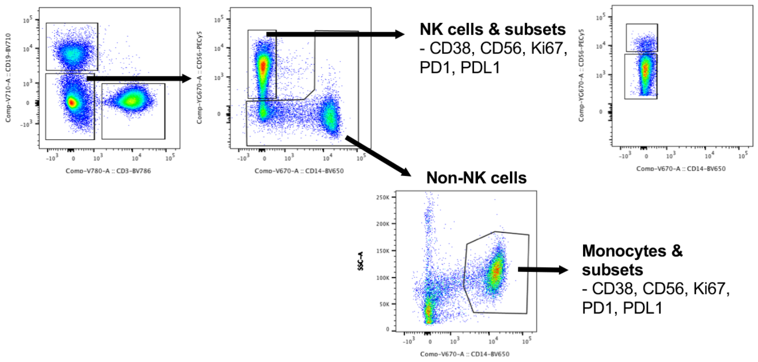

Figure 3. Gating NK cells and subsets

Mixed/ myeloid panel
CD3
CD14
CD19
CD56
CD16
CD11c
HLADR
CD27
CD11b
CD123
CD141
CD1c
CD15
CD244
FVS

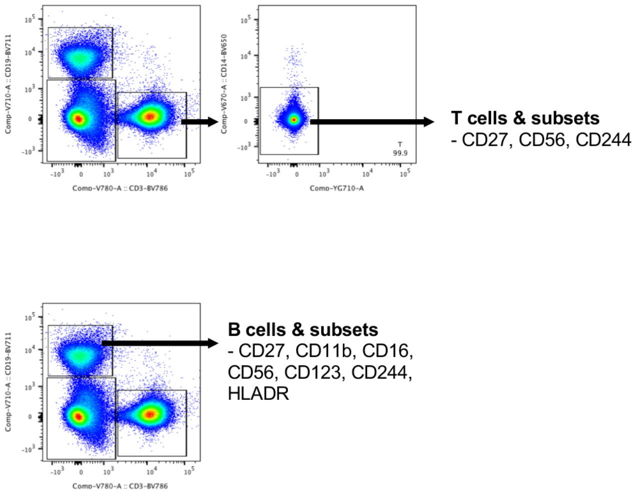

Figure 4. Gating mixed myeloid panel from CD3|CD19

Mixed/ myeloid panel
CD3
CD14
CD19
CD56
CD16
CD11c
HLADR
CD27
CD11b
CD123
CD141
CD1c
CD15
CD244
FVS

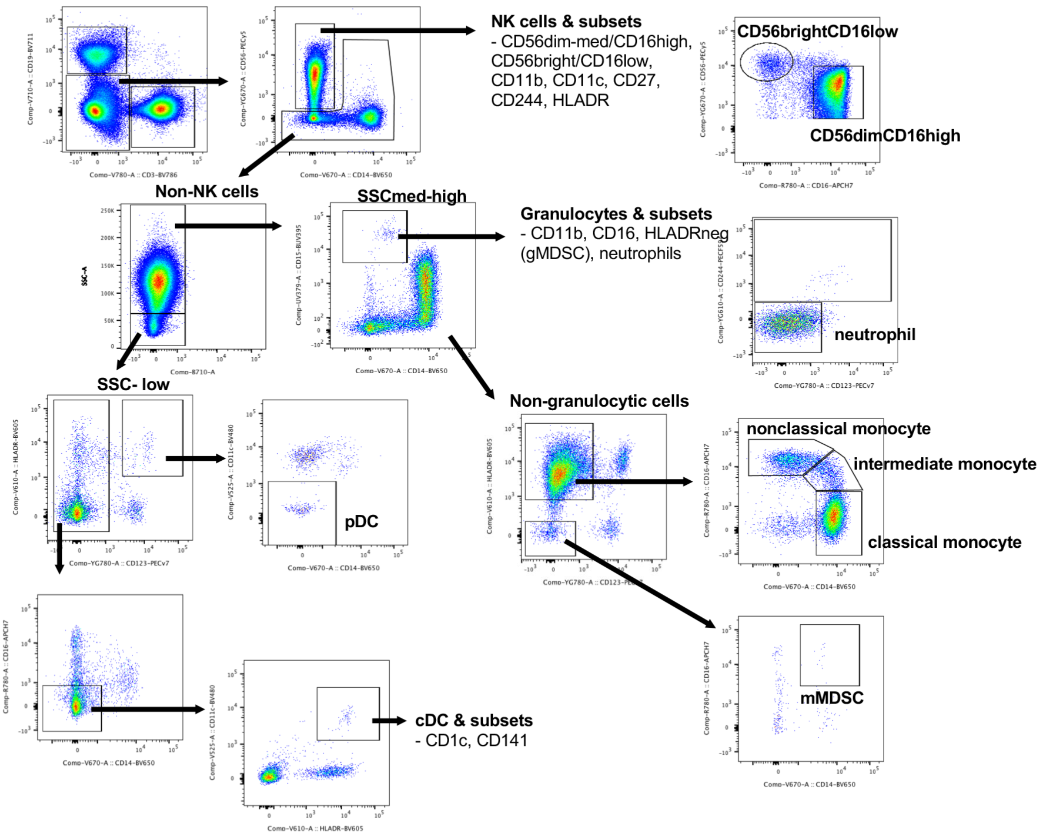

Figure 5. Gating subsets

Cytokines (stimulated + resting cells)
CD3
CD14
CD19
CD56
CD4
CD8
~~CD16~~
Va24Ja18
CD11c
CD11b
Va7.2
TCRγδ
IL2
IFNγ
TNFα
FVS

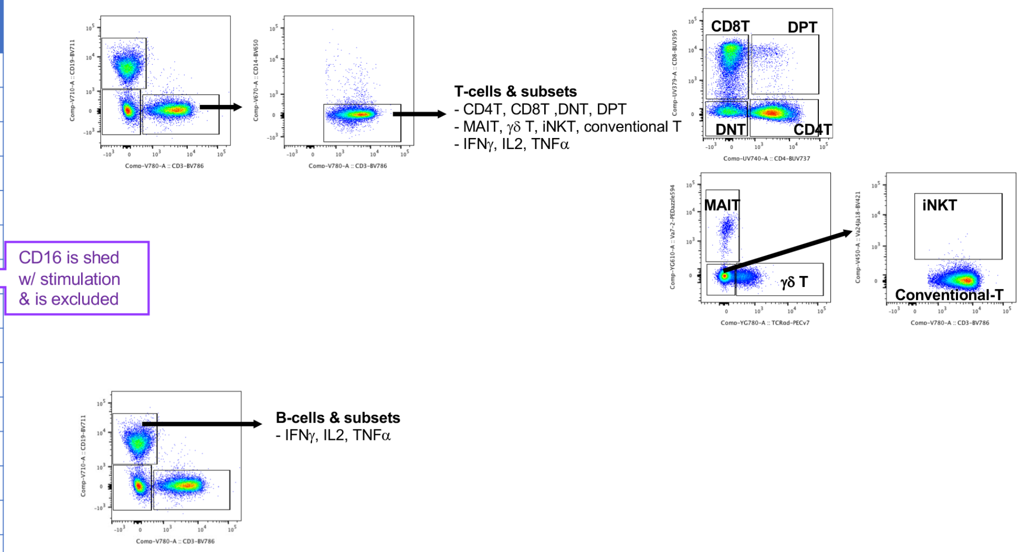

Cytokines (stimulated + resting cells)
CD3
CD14
CD19
CD56
CD4
CD8
~~CD16~~
Va24Ja18
CD11c
CD11b
Va7.2
TCRγδ
IL2
IFNγ
TNFα
FVS

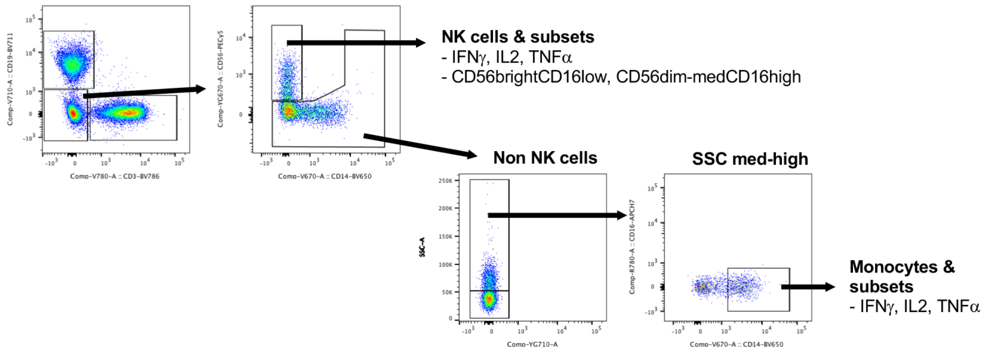

Figure 6. Cytokine/stimulated resting panel

Appendix 2. Patient disposition for MoST screening to trial enrollment

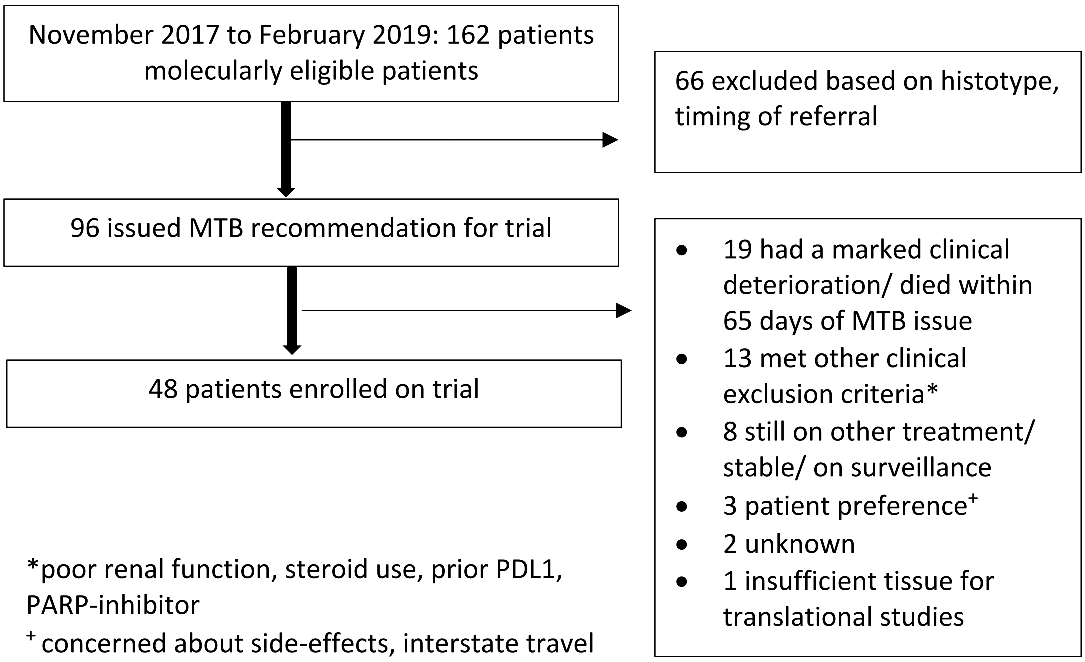

*poor renal function, steroid use, prior PDL1, PARP-inhibitor

+ concerned about side-effects, interstate travel

Appendix 3. Details of time to progression (TTP) prior to treatment and calculated TTP ratios

Patient ID	TTP1	TTP2 (on trial)	TTP2/TTP1 ratio	Prior treatment details
**Group 1**				
D003	4.07	2.13	0.52	**adjuvant** cisplatin + doxorubicin
D012	5.90	4.33	0.73	pazopanib
D018	16.27	33.80	2.08	**adjuvant** cisplatin + gemcitabine
D019	2.97	3.57	1.20	taxol
D026	3.53	14.50	4.10	Carboplatin + paclitaxel + avastin
D038	3.07	0.67	0.22	palbociclib
D040	6.17	10.03	1.63	irinotecan + Temozolomide
D041	1.00	7.47	7.47	gemcitabine (maintenance)
D042	8.67	0.23	0.03	temozolamide
D043	13.00	1.80	0.14	FOLFIRI
D044	3.30	2.60	0.79	doxorubicin
D045	1.87	3.70	1.98	FOLFIRI + Bevacizumab
D046	4.80	1.80	0.38	FOLFIRINOX
D047	15.17	22.60	1.49	FOLFOXIRI
D048	1.07	3.90	3.66	**adjuvant** carboplatin + paclitaxel
D049	2.33	0.53	0.23	Cisplatin + Gemcitabine
**Group 2**				
D001		9.13		**no prior treatment**
D002	4.43333	5.47	1.23	**adjuvant** oxaliplatin, capecitabine / F-FU
D006	8.06667	3.43	0.43	alternating IV avastin and IT cytarabine + etopside
D007	2.66667	35.87	13.45	Zometa, Zoladex & Aromasin
D008	4	12.10	3.03	gemcitabine
D011	2.8	3.57	1.27	Votrient
D013		10.70		carboplatin+paclitaxel x 3 cycles
D015	2.03333	3.47	1.70	raltitrexed
D016	6.06667	3.60	0.59	procarbazine + lomustine + vincristine
D017	12.1667	17.97	1.48	CDE clinical trial
D020	24.3667	3.67	0.15	Cediranib
D021	1.16667	16.13	13.83	cetuximab
D023		4.47		Avastin
D024	3.33333	3.63	1.09	palbociclib
D027	5.86667	6.73	1.15	FOLFOX
D029	1.7	30.57	17.98	**adjuvant** carboplatn + pacli x 6 cycles
D030	5.1	30.30	5.94	carboplatin + paclitaxel
D032	19.2333	7.17	0.37	cisplatin + Doxorubicin + Cycophosphamide
D033	4.9	8.20	1.67	palbociclib + Letrazole
D034	7.83333	12.87	1.64	paclitaxel
D037	5.8	28.43	4.90	**adjuvant** cisplatin + doxorubicin + HD-MTX
D004	0.53333	1.53	2.88	TAC
D005	4.96667	1.60	0.32	temozolomide
D009	7.53333	1.60	0.21	LXS196 a protein Kinase C
D010	3.5	1.67	0.48	FOLFOX

D014	8.4	1.63	0.19	FOLFIRI
D022	3.06667	1.67	0.54	letrozole
D025	3.73333	1.90	0.51	eribulin
D031	1.36667	1.70	1.24	cisplatin + etoposide
D035	4.2	1.80	0.43	lenvatinib
D036	6.86667	1.73	0.25	liposomal irinotecan + 5-fluorouracil (4 cycles)
D039	24.8667	1.70	0.07	dendritic cell + NKC immunotherapy trial

Appendix 4A. Adjudicated SAEs related to any substudy treatment (definitely, probably, possibly)

Patient ID	System Organ Class	Event term	Grade	Start date - end date	Related/ expected olaparib*	Related/expected durvalumab*
D015	Blood disorders	anemia	3	28/02-02/03	related/ expected	unrelated / -
	Blood disorders	anemia	4	01/05-03/05	related/ expected	unrelated / -
D021	Renal disorders	acute kidney injury	3	01/07-06/07	related /expected	unrelated / -
	Renal disorders	acute renal failure	3	21/01-23/01	related /expected	related /expected
D025	Gastrointestinal disorders	abdominal pain	3	15/04-19/04	related /expected	unrelated / -

SAEs – serious adverse events. Note: '-/-' & ' -/-' indicates not possible to specify relatedness.

Appendix 4B. Listing adjudicated SAEs unrelated to any substudy treatment (unlikely, not related)

Patient ID	System Organ Class	Event term	Grade	Start date - End date	Related/Expected Olaparib *	Related/Expected Durvalumab *
D008	Metabolism disorders	Hyperglycemia	3	22/11/2018 - 25/11/2018	- / -	- / -
D010	Infections and infestations	Lung infection	3	22/01/2018 - 29/01/2018	- / -	- / -
	Gastrointestinal disorders	Pancreatitis	3	01/02/2018 - 08/02/2018	- / -	- / -
D018	Renal and urinary disorders	Hematuria	3	09/01/2019 - 10/01/2019	- / -	- / -
	Vascular disorders	Hypotension	3	03/04/2019 - 05/04/2019	- / -	- / -
D021	Investigations	Alanine aminotransferase increased	4	07/06/2019 -	- / -	- / -
D024	Respiratory, thoracic, and mediastinal disorders	Other: Community Aquired Pneumonia	4	29/05/2018 - 06/06/2018	- / -	- / -
D032	Gastrointestinal disorders	Colonic perforation	3	26/11/2018 - 21/12/2018	- / -	- / -
D041	Eye disorders	Blurred vision	3	19/11/2018 - 20/11/2018	- / -	- / -
D049	Infections and infestations	Sepsis	5	02/03/2019 -	- / -	- / -

SAEs – serious adverse events. Note: '-/-' &' -/-' indicates not possible to specify relatedness.

Appendix 5. Confirmed germline alterations

Patient ID	Cancer type	Gene with germline variant	ClinVar
D025	Myxoid liposarcoma	*NBN*	pathogenic
D032	Breast, IDC	*ATM*	pathogenic
D034	Papillary thyroid carcinoma	*ATM, NBN*	likely pathogenic, pathogenic/ likely pathogenic
D035	Pancreas adenocarcinoma	*ATM*	pathogenic
D041	Glioma	*BRCA2 c.5909C>A*	pathogenic
D045	Pancreas adenocarcinoma	*BRCA2 c.7975A>G*	pathogenic
D046	Rectal adenocarcinoma	*BRCA2 c.7988A>T*	pathogenic
D048	Pancreas adenocarcinoma	*BRCA2 c.5238dupT*	pathogenic

IDC – infiltrating ductal carcinoma

Appendix 6 – Forest plots

6A. Forest plot of progression-free survival for biological and clinical factors by group

Group 1

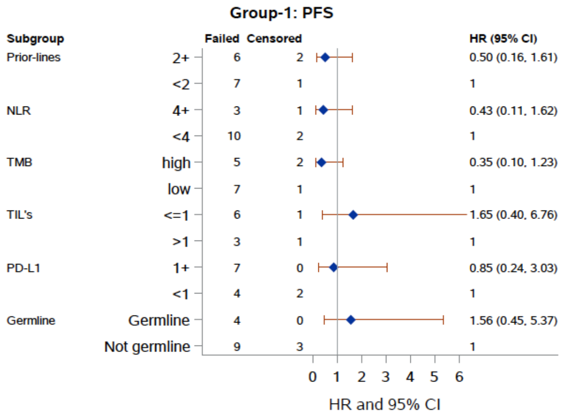

Group 2

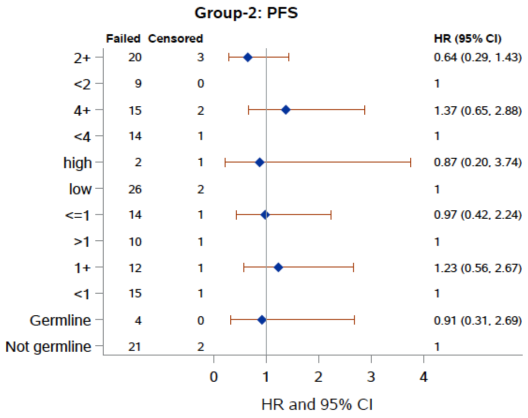

Group 1 – Patients with BRCA1/2 alterations. Group 2 – Patients with other homologous recombination repair (HRR) alterations. None of the clinical factors examined – number of prior lines of therapy, NLR, TMB, TILs, PDL-1 status or germline versus somatic demonstrated a significant difference in PFS. Abbreviations: CI – confidence interval, HR – hazard ratio for death, NLR – neutrophil-lymphocyte ratio, PFS – progression-free survival, TILs – tumour infiltrating lymphocytes, TMB – tumour mutational burden.

6B. Forest plot of overall survival for biological and clinical factors by group

Group 1

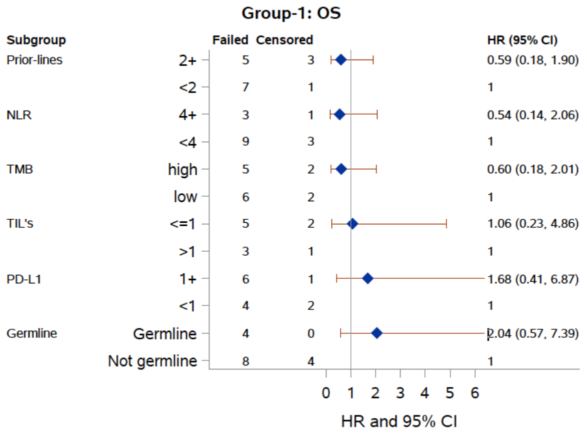

Group 2

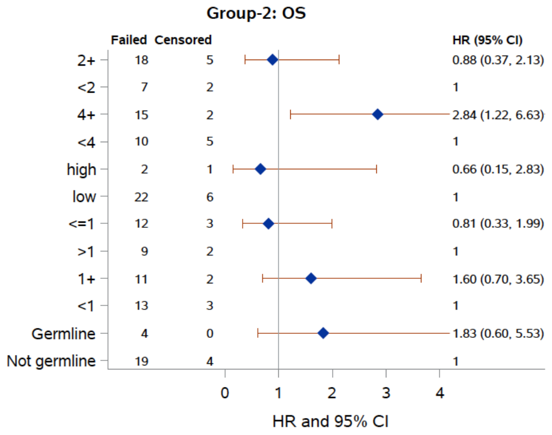

Group 1 – Patients with BRCA1/2 alterations. Group 2 – Patients with other homologous recombination repair (HRR) alterations. Most clinical factors examined – number of prior lines of therapy, TMB, TILs, PDL-1 status or germline versus somatic demonstrated no significant difference in OS. Only a high NLR ≥4 in group 2 was associated with a worse OS. Abbreviations: CI – confidence interval, HR – hazard ratio for death, NLR – neutrophil-lymphocyte ratio, OS – overall survival, TILs – tumour infiltrating lymphocytes, TMB – tumour mutational burden.

6C. Forest plot of progression-free survival and overall survival for biological and clinical factors for the **overall** trial population

Progression-free survival

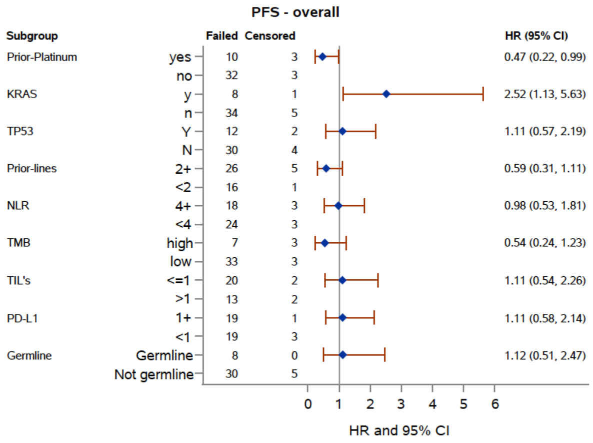

Overall survival

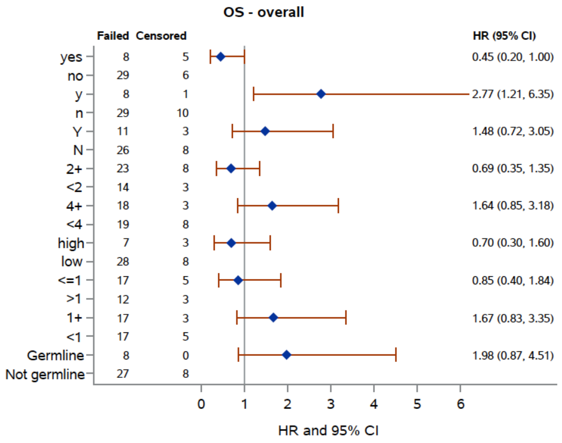

Progression-free and overall survival for the overall trial population, including both groups (*BRCA1/2* as well as patients with other *HRR alterations*). The presence of a *KRAS* mutation was associated with a worse progression-free and overall survival. Abbreviations: CI – confidence interval, HR – hazard ratio for death, NLR – neutrophil-lymphocyte ratio, OS – overall survival, PFS – progression-free survival, TILs – tumour infiltrating lymphocytes, TMB – tumour mutational burden.

Appendix 7

Table 7A. Flow cytometry data, immune cell subsets at baseline, week 4 and 8 that were associated with good responders (durable response is ≥ 2 years OS and ≥ 6 months PFS) versus poor responders

PBMC phenotype	Defining markers	Observation	Fold Change	*P-value*
**Week 0 (Baseline)**				
[Convtl T cell all] IFNγ+	CD3+CD19- CD14-> Va7.2- TCRγδ- Va24Ja18- IFNg+	more	**1.25**	*0.154*
[CD8+ T-cell all]/IFNγ+	CD3+CD19- CD14- > CD4-CD8+> IFNγ+	more	**1.45**	*0.135*
[Convt CD8+ T-cell]+ IFNγ+/all *	CD3+CD19- CD14- > CD4-CD8+> Va7.2- TCRgd- Va24Ja18- IFNγ+	more	**1.51**	*0.154*
[γδ T-cells] CD4+	CD3+CD19- CD14- > Va7.2- TCRγδ+> CD4+	less	**0.55**	*0.135*
[γδ T-cells] IL2+	CD3+CD19- CD14- > Va7.2- TCRγδ+> IL2+	less	**0.44**	*0.135*
B-cell CD38+	CD3-CD19+> CD38+	more	**1.49**	*0.0935*
B-cell CD38+ high	CD3-CD19+> CD38high	more	**2.59**	*0.0385*
NK cells/all	CD3-CD19- CD56+CD14-	less	0.61	*0.135*
NK all CD56dim CD16high NK/all	CD3-CD19- CD56+CD14-> CD56dim CD16high	less	**0.54**	*0.135*
NK all CD16+	CD3-CD19- CD56+CD14-> CD16+	less	**0.54**	*0.135*
[NK] TNFα+	CD3-CD19- CD56+CD14-TNFα+	less	**0.45**	*0.137*
Central memory DNT	T-cell > CD4-CD8- > CD45RA-CCR7+	more	**3.28**	*0.145*
DPT Ki67+	CD3+CD19- CD14 > CD4+CD8+> Ki67+	less	**0.52**	*0.135*
DPT all CD25+CD56+	CD3+CD19- CD14-> CD4+CD8+> CD25+ CD56+		**0.23**	*0.135*
**Week 4 (+ olaparib)**				
Exhausted T-cell	CD3+CD19- CD14-> CD244low-med/T	less	**0.62**	*0.201*
[All T-cells] IL-2+	CD3+CD19- CD14-> IL-2+	more	1.04	*0.201*
T-cell CD4+ activated	CD3+CD19- CD14- > not-iNKT > CD4+CD8-> CD38+, > Ki67+	more	1.48	*0.201*
[γδ T-cells] TNFα+	CD3+CD19- CD14- > Va7.2- TCRgδ + TNFα+	more	1.56	*0.201*
B-cell CD38+	CD3-CD19+> CD38+	more	1.35	*0.201*
B-cell CD38+ high	CD3-CD19+> CD38high	more	2.07	*0.201*
DNT cells CD56+	CD3+CD19- CD14-> CD4-CD8-CD56+	less	0.47	*0.201*
DPT cells CD25+	CD3+CD19- CD14-> CD4-CD8-CD25+	more	1.41	*0.201*
Monocytes All	CD3-CD19- SSCmed-high CD123-HLADR+	less	0.72	*0.201*
All classical monocytes	CD3-CD19- SSCmed-high CD123-HLADR+ >CD14+CD16-	less	0.7	*0.201*
**Week 8 (+olaparib and durvalumab)**				
All T-cells IFNγ+	CD3+CD19- CD14-> IFNγ+	more	3.2	*0.112*
All T-cells TNFα+	CD3+CD19- CD14-> TNFα+	more	8.6	*0.124*
Activated CD8+ cells T-reg	CD8+ T cell > CD25+CD127-> Ki67+	more	1.89	*0.112*
B-cell CD38+	CD3-CD19+> CD38+	more	1.33	*0.112*
B-cell CD38+ high all	CD3-CD19+> CD38high	more	3.01	*0.112*
NK TNFα+	CD3-CD19- CD56+CD14-TNFα+	more	2.89	*0.112*
DNT cells CD56+	CD3+CD19- CD14-> CD4-CD8-CD56+	less	0.508	*0.112*
Monocytes HLA DR high	CD3-CD19- SSCmed-high CD123-HLADR+	more	1.88	*0.112*
All Neutrophils	CD3-CD19- SSCmed-high CD15highCD14-> CD244- CD123-	less	0.69	*0.112*

Evaluable samples week 0 (9 good/29 poor), week 4 (8 good/28 poor), week 8 (8 good/ 21 poor). Only immune cell populations that were significant using Mann Whitney U test ($P \leq 0.05$) are shown. Furthermore, *P-values* have been adjusted using the Benjamini-Hochberg method for multiple test correction(). [ ] denotes panel 3 where cells were primed with PMA and ionomycin.

Table 7B. Immune cell subsets that were associated with better overall survival and/or progression free survival at each timepoint

PBMC phenotype	Defining markers	OS	*P-value*	PFS	*P-value*
**Week 0 (*Baseline*)**					
All T-cells	CD3+CD19- CD14-	more	*0.0013*		
[γδ T-cells] IL2+	CD3+CD19- CD14- > Va7.2- TCRγδ+> IL2+	less	*0.0053*	less	*0.0006*
B-cell CD38+	CD3-CD19+> CD38+	more	*0.004*	more	*0.0006*
B-cell CD38+ high	CD3-CD19+> CD38high	more	*0.0077*	more	*0.0003*
B-cell PD-L1+	CD3-CD19+> PD-L1+	more	*0.008*	more	*0.0002*
[NK] TNFα+	CD3-CD19- CD56+CD14-TNFα+	less	*0.0013*	less	*0.0005*
DPT all CD25+CD56+	CD3+CD19- CD14-> CD4+CD8+> CD25+ CD56+			less	*0.0053*
Week 4 (+ olaparib)					
[All T-cells] IL-2+	CD3+CD19- CD14-> IL-2+	*more*	*0.0006*		
[γδ T-cells] IL2+	CD3+CD19- CD14- > Va7.2- TCRγδ+> IL2+			*less*	*0.0275*
**Week 4 (*+ olaparib and durvalumab*)**					
All B-cells	CD3-CD19+	more	***0.0055***		
B-cell CD38+ high	CD3-CD19+> CD38high	more	***0.0165***		
Monocytes HLA DR high	CD3-CD19- SSCmed-high CD123-HLADR+	more	***0.0311***		

Differences in survival were calculated by Mantel Cox rank test. *P-values* from higher ≥ median levels of immune subsets. Furthermore, *P-values* that survived multiple comparisons using the Benjamini-Hochberg method, (adjusted P-values) are shown. [ ] denotes panel 3 where cells were primed with PMA and ionomycin.

Appendix 8. Comparative analysis of immune cell populations following treatment with olaparib at week 4.

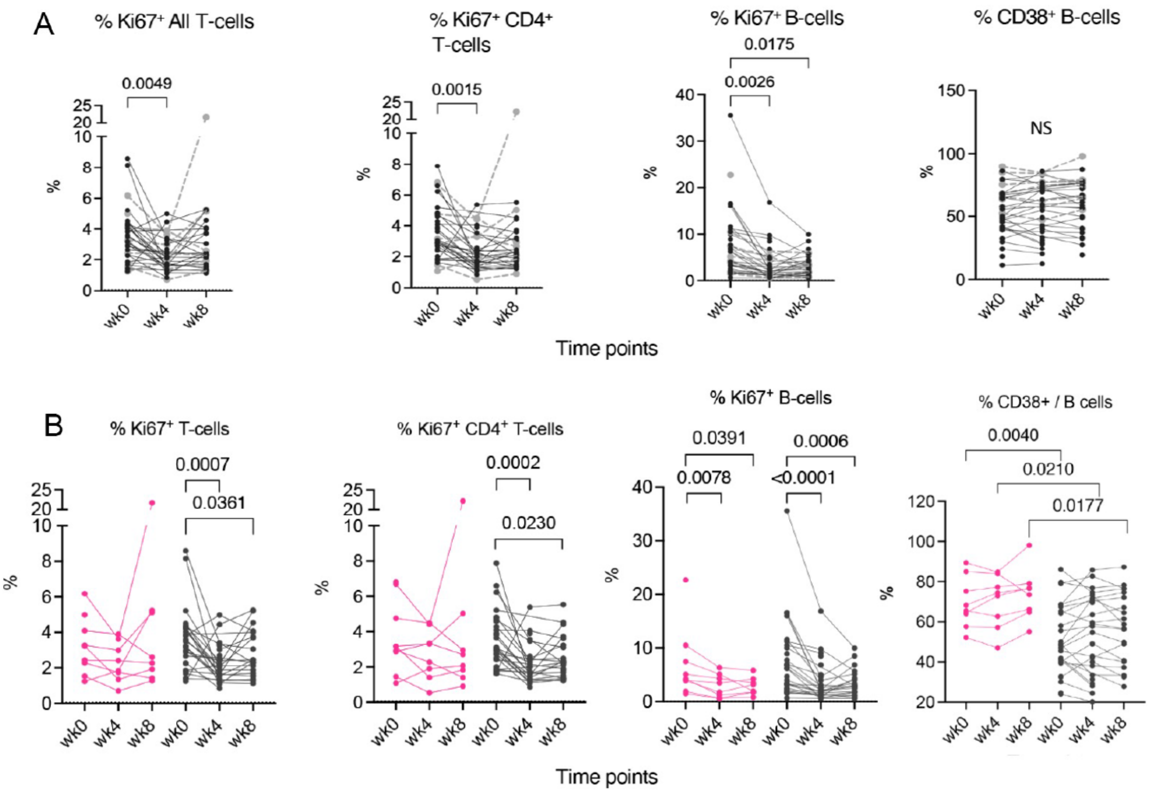

Fig 8. Comparative analysis of immune cell populations following treatment with olaparib at week 4

A) Showing lymphoid populations that significantly changed from baseline to week 4 in all patients, with the exception of CD38+ B cells.

B) Analysis of populations segregated into good responders (pink) and poor responders. Kruskal-Wallis test was conducted for multiple comparisons.

Appendix 9A. Comparison of immune cell phenotype in patients with BRCA1/2 versus all other HRR alterations at baseline and at week 4 following olaparib.

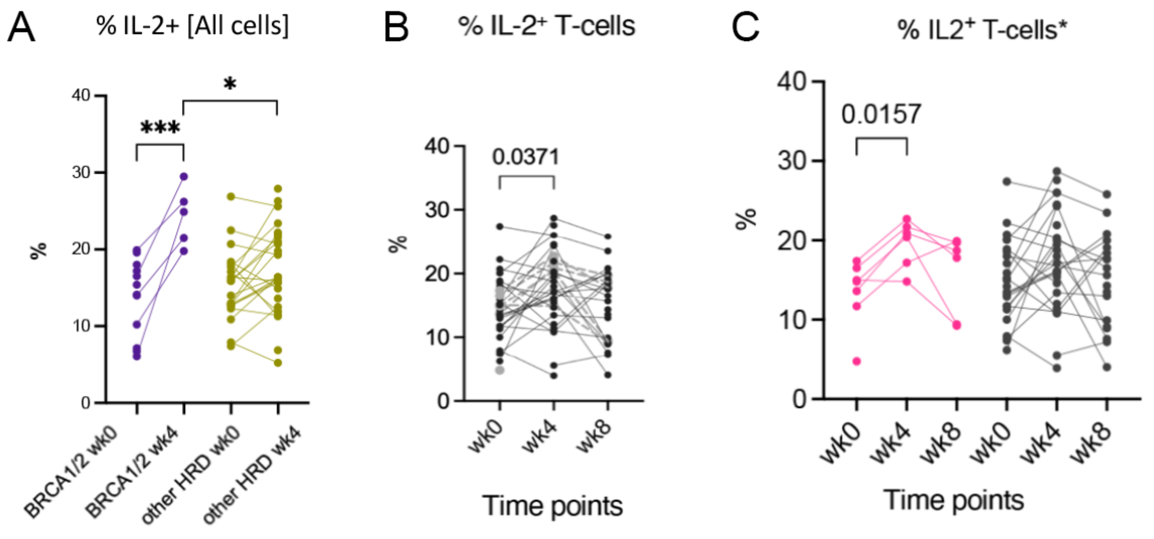

Figure 9A. Single live cells shown. B) increased IL-2 expression seen at week 4 following olaparib. C) demonstrates correlation with good responders.

Appendix 9B. Comparative analysis of immune cell populations following treatment with both olaparib and durvalumab at week 8.

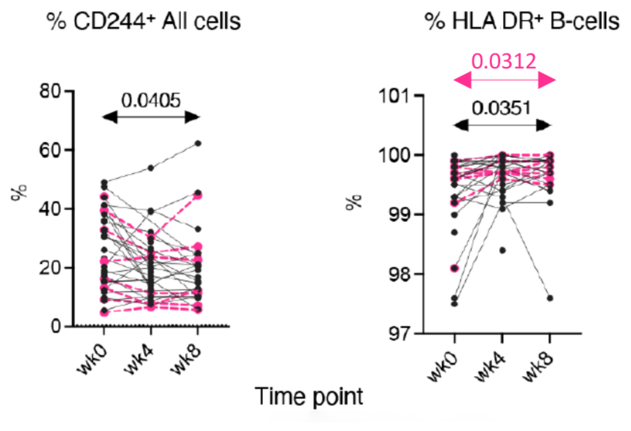

Figure 9B Showing immune cell populations that most significantly changed after O+D at 8 weeks (black lines), Good responders are shown in pink. Kruskal-Wallis test was conducted for multiple comparisons.
